# Examining the Association of Socioeconomic Position with Microcephaly and Delayed Childhood Neurodevelopment among Children with Prenatal Zika Virus Exposure

**DOI:** 10.3390/v12111342

**Published:** 2020-11-23

**Authors:** Grace M. Power, Suzanna C. Francis, Nuria Sanchez Clemente, Zilton Vasconcelos, Patricia Brasil, Karin Nielsen-Saines, Elizabeth B. Brickley, Maria E. Moreira

**Affiliations:** 1 Department of Disease Control, Faculty of Infectious and Tropical Diseases, London School of Hygiene & Tropical Medicine, London WC1E 7HT, UK; grace.power@bristol.ac.uk; 2 Department of Infectious Disease Epidemiology, London School of Hygiene & Tropical Medicine, London WC1E 7HT, UK; suzanna.francis@lshtm.ac.uk (S.C.F.); nuria.sanchez-clemente@lshtm.ac.uk (N.S.C.); 3 MRC Integrative Epidemiology Unit, Population Health Sciences, Bristol Medical School, University of Bristol, Bristol BS8 2BN, UK; 4 Fundação Oswaldo Cruz, Rio de Janeiro 21040-900, Brazil; zilton.vasconcelos@iff.fiocruz.br (Z.V.); patricia.brasil33@gmail.com (P.B.); 5 Pediatrics, University of California, Los Angeles, CA 90095, USA; knielsen@mednet.ucla.edu

**Keywords:** Zika virus, congenital Zika syndrome, microcephaly, neurodevelopment, socioeconomic position, health equity

## Abstract

Increased rates of Zika virus have been identified in economically deprived areas in Brazil at the population level; yet, the implications of the interaction between socioeconomic position and prenatal Zika virus exposure on adverse neurodevelopmental outcomes remains insufficiently evaluated at the individual level. Using data collected between September 2015 and September 2019 from 163 children with qRT-PCR and/or IgM-confirmed prenatal exposure to Zika virus participating in a prospective cohort study in Rio de Janeiro, Brazil (NCT03255369), this study evaluated the relationships of socioeconomic indicators with microcephaly at birth and Bayley-III neurodevelopmental scores during the early life course. Adjusted logistic regression models indicated increased odds of microcephaly in children born to families with lower household income (OR, 95% CI: 3.85, 1.43 to 10.37) and higher household crowding (OR, 95% CI: 1.83, 1.16 to 2.91), while maternal secondary and higher education appeared to have a protective effect for microcephaly compared to primary education (OR, 95% CI: 0.33, 0.11 to 0.98 and 0.10, 0.03 to 0.36, respectively). Consistent with these findings, adjusted linear regression models indicated lower composite language (−10.78, 95% CI: −19.87 to −1.69), motor (−10.45, 95% CI: −19.22 to −1.69), and cognitive (−17.20, 95% CI: −26.13 to −8.28) scores in children whose families participated in the Bolsa Família social protection programme. As such, the results from this investigation further emphasise the detrimental effects of childhood disadvantage on human health and development by providing novel evidence on the link between individual level socioeconomic indicators and microcephaly and delayed early life neurodevelopment following prenatal Zika virus exposure.

## 1. Introduction

Zika virus (ZIKV) is a mosquito-borne flavivirus [1,2], principally transmitted by the *Aedes aegypti* vector. *Ae. aegypti* is an anthropophilic mosquito species with a high daily survival rate, capable of facilitating explosive arboviral epidemics in urban settings [3]. Vertical transmission of ZIKV during pregnancy has been associated with adverse developmental consequences in infected offspring, including microcephaly and other neurological impairments, which are collectively recognised as congenital Zika syndrome (CZS) [4].

Studies have demonstrated increased frequencies of ZIKV infection and CZS in economically deprived areas of Brazil at the population level [5,6]. While these studies play an important role in assessing the association between ZIKV and social conditions, the existing evidence base relies on ecological study designs with a geographically defined group as the unit of observation. Not only is ecological fallacy a potential limitation for interpreting associations, but socioeconomic risk factors at the individual level remain insufficiently identified and evaluated. In addition to congenital ZIKV infection, socioeconomic position (SEP) may also influence neurodevelopment. Children typically experience poorer health and developmental outcomes with higher levels of disadvantage [7]. Risk factors for cognitive and socioemotional developmental delays have been shown to include nutrient deficiencies and social and economic deprivation, whilst known protective factors comprise breastfeeding and maternal education [8].

This study investigates the associations between socioeconomic factors and two outcomes, microcephaly and neurodevelopmental delays, in a cohort of 163 infants with in utero ZIKV exposure, in the State of Rio de Janeiro, Brazil. We hypothesise that there may be a relationship between measures indicative of lower SEP and adverse neurodevelopmental outcomes among infants with prenatal exposure to ZIKV. A better understanding of the most at-risk groups in the event of a future ZIKV outbreak could help to drive policy solutions that encourage more targeted approaches to public health interventions aimed at reducing health and developmental inequities.

## 2. Materials and Methods

A prospective cohort study (ZIKAIFF) was conducted between September 2015 and September 2019 at Instituto Nacional de Saúde da Mulher, da Criança e do Adolescente Fernandes Figueira (IFF/Fiocruz), a reference hospital for women, children and adolescents in Rio de Janeiro, Brazil. Mothers of children in the cohort provided informed written consent for their children to participate. Local ethical approval was obtained for the protocol of the original cohort study titled “Exposição Vertical ao Zika Virus e suas conseqüências no neurodesenvolvimento da criança/Vertical Exposure to Zika Virus and Its Consequences for Child Neurodevelopment: Cohort Study in Fiocruz/IFF” by the Oswaldo Cruz Foundation (Fiocruz), Rio de Janeiro, Brazil (Plataforma Brasil, CAAE: 52675616.0.0000.5269 and registered in ClinicalTrials.gov (NCT03255369). Ethical approval for this project was granted on 10 May 2019 by the London School of Hygiene & Tropical Medicine MSc Research Ethics Committee (Ref: 15951).

### 2.1. Study Population

Participants in the ZIKAIFF cohort comprised children born between February 2016 and September 2017 [9], in the State of Rio de Janeiro, Brazil. The cohort included children with suspected prenatal ZIKV exposure, identified from two key sources: (i) those born to symptomatic women presenting with rash during pregnancy and (ii) those born to women referred to IFF/Fiocruz due to foetal abnormalities during pregnancy detected through ultrasound screening.

The present investigation was limited to the 163 livebirths (55.1%; 163/296 of the full cohort) with lab-confirmed prenatal exposure to ZIKV that participated in infant clinical assessment at birth, and if normocephalic, at least one subsequent neurodevelopmental evaluation with the Bayley-III instrument. For this analysis, in utero ZIKV exposure was confirmed through either (i) the detection of ZIKV RNA by qRT-PCR testing of maternal serum, urine, amniotic fluid, breast milk, and/or placenta samples or neonatal serum, urine and cerebrospinal fluid sample [10] or (ii) through the detection of IgM in neonatal serum samples using the Centers for Disease Control and Prevention (CDC) Zika IgM antibody capture enzyme-linked immunosorbent assay (MAC-ELISA) [11].

### 2.2. Exposures

Several primary exposures were investigated as potential socioeconomic risk markers at the individual level. These included maternal educational level (partial/completed higher, secondary or primary school education), maternal race/ethnicity (White Brazilians, mixed-race Afro-Brazilians, Black Afro-Brazilians and East Asian Brazilians), household monthly income (relative to the 2019 minimum wage of BRL998) (Classes A, B, C and D: >2× minimum wage and Class E: <2× minimum wage), household participation in the Bolsa Família conditional cash transfer social protection program (yes or no) and household crowding index (individuals in the house/bedrooms in the house), obtained from parent-reported data through survey questionnaires given to caregivers by clinical staff at IFF/Fiocruz in Rio de Janeiro, Brazil, upon enrolment in the cohort.

### 2.3. Outcomes

#### 2.3.1. Infant Clinical Assessments

The outcome variable of microcephaly was defined as a head circumference Z score of more than two standard deviations (SD) below the mean for gestational age and sex, consistent with the latest Brazilian Ministry of Health’s case definition. Head circumference measurements were taken from all live newborns and evaluated using the INTERGROWTH-21st Global Perinatal Package [12]. All evaluations took place at IFF/Fiocruz in Rio de Janeiro, Brazil by paediatric specialists at birth.

#### 2.3.2. Neurological Evaluations

Bayley-III assessments were offered to all normocephalic children. Bayley-III assessments of children born with microcephaly were not routinely undertaken as the functional challenges faced by children in this group precluded further assessment of developmental milestones using this instrument [13,14]. Bayley-III is an internationally accepted instrument used to assess the development of infants and young children aged between 1 and 42 months. The assessment and training materials have been translated into Brazilian Portuguese and validated for use in Brazil [15].

The Bayley-III scales derive a developmental quotient by evaluating three domains: the language scale, which assesses expressive and receptive language; the motor scale, which assesses fine and gross motor skills; and the cognitive scale [16]. Composite scores were obtained for each subset to determine performance compared with the normative population and presented as a continuous variable. They were scaled to a metric, with a mean of 100, SD of 15 and range of 40 to 160. Developmental delay was defined as “at risk” if performance was between 1 and 2 SD below the mean (i.e., a score of 70–85) and “severely delayed” if the score was more than 2 SD below the mean (i.e., a score <70).

For children who underwent repeated evaluations, the Bayley-III scores obtained at the oldest age were used in the current analysis. Assessments took place at IFF/Fiocruz and were performed by trained psychologists.

### 2.4. Additional Covariates

**Figure 1:**
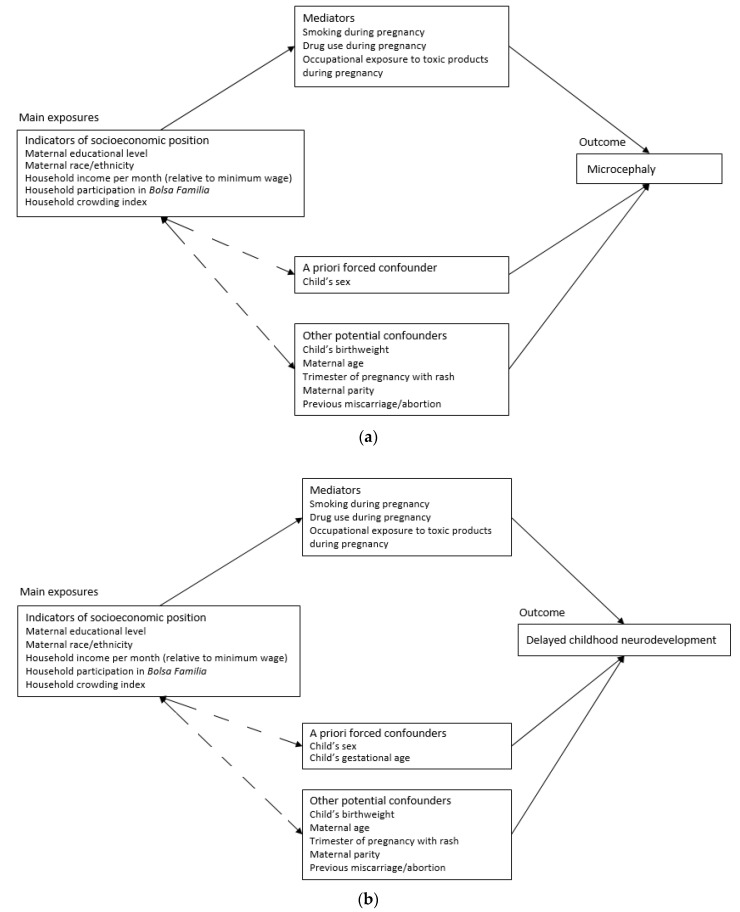
Conceptual frameworks for the association between the exposures of interest; social determinants and the outcomes; microcephaly (**a**) and delayed neurodevelopment (**b**) following in utero exposure to ZIKV.

Further potential confounders and effect modifiers were considered and integrated into conceptual hierarchical frameworks (Figure 1). These were derived from the literature and through conversations with clinical staff at IFF/Fiocruz [17].

Since being a recipient of BF is conditional on having a low-income as well as school attendance, health monitoring and prenatal care attendance, household participation in BF was not considered a confounder when household income was the main exposure, as it was assumed on the causal pathway [18]. The variables indicating smoking, drug-taking and occupational exposure to toxic products during pregnancy, which may be influenced by SEP, were assumed on the causal pathway between SEP indicators and both outcome measures. Moderate exposure to maternal smoking, drug-taking and toxic products can impact foetal brain development and may consequently be risk factors for microcephaly and neurodevelopmental delays [19] and thus were not included in the multivariable analyses.

### 2.5. Data Cleaning and Missing Values

Data entry errors were checked as part of the quality assessment. Duplicates were removed and outliers queried and rectified at the study site. Missing data were explored and missingness patterns investigated (Table A1). The complete-case analysis was employed in final multivariable models.

### 2.6. Statistical Analysis

Multivariate regression analyses were conducted (logistic models for assessing microcephaly and linear models for the three continuous composite Bayley-III score outcomes: language, motor and cognitive). The variable indicating child’s sex was a priori forced into each of the multivariate models. Gestational age was a priori forced into multivariate linear models for continuous Bayley-III score outcomes.

Conceptual hierarchical frameworks aided in the determination of mediators and confounders when fitting models. To estimate the effect of individual level SEP risk factors on the odds of microcephaly, a forward selection approach was used. Primary exposure variables indicating SEP were used to initiate each model with the forced variables that were a priori determined. Potential confounders, including SEP variables that were not considered the main exposure of interest in that particular model, were then built into each model according to how much their inclusion in the model changed the effect estimate for the main exposure. Variables were added to models only if they changed the effect estimate by more than 10%. To avoid problems of data sparsity, models contained no more than five parameters, since there were 51 events of microcephaly. Multiple linear regression models were then fitted for each of the composite Bayley-III score outcomes with SEP exposure variables, the forced variables selected a priori and the strongest potential confounding variables, ensuring that there were at least five observations for each variable added to the model to mitigate sparse data bias. To assess consistency regarding direction and magnitude of estimates, sensitivity analyses were performed using data with participants with suspected prenatal exposure to ZIKV who did not have qRT-PCR or IgM confirmation. This dataset was larger (*n* = 286) and had 91 microcephaly cases. Data analysis was performed in Stata, version 13.0 (StataCorp., College Station, TX, USA)

## 3. Results

**Figure 2:**
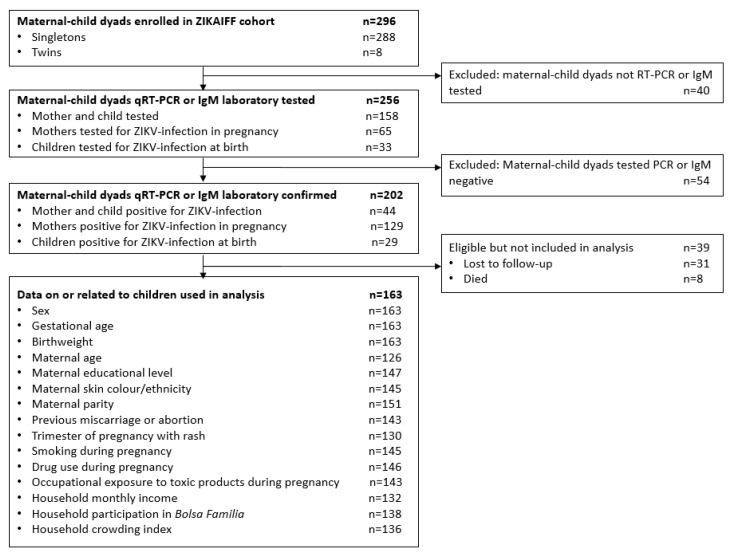
Flow diagram for cohort selection based on study inclusion and exclusion criteria.

Of the 296 maternal-child dyads enrolled in the ZIKAIFF cohort, 256 (86.5%; 256/296) were qRT-PCR or IgM laboratory tested and 202 (68.2%; 202/296) had qRT-PCR (85.6%; 173/202) or IgM (36.1%; 73/202) confirmation for ZIKV. Of the confirmed cases, eight (4.0%; 8/202) of the children died prior to outcome ascertainment and 31 (15.3%; 31/202) of the normocephalic children were lost to follow-up. In total, 163 (55.1%; 163/296) participants of the total cohort were included in the final study sample (Figure 2).

The study sample comprised 84 (51.5%) females and 79 males (48.5%). Fifty-one (31.3%) had microcephaly at birth and 112 were normocephalic at birth. Children born with a normal head circumference were followed up with their last Bayley-III neurodevelopmental assessment performed at a median (IQR) age of 19.6 months (range: 4.9 to 40.1 months). The median (IQR) gestational age at delivery was 38 weeks (38–40 weeks) and birthweight was 3060 g (2675–3420 g). In total, 18.4% (30/163) had low birthweight (<2500 g). Mothers were aged between 17 and 43 years and lived in the State of Rio de Janeiro, Brazil at the time of enrolment. Amongst those with data collected on maternal education, 15.0% (22/147) of children were born to mothers with up to primary school education, 52.4% (77/147) with some or completed secondary school education and 32.7% (48/147) with some or completed higher education. Furthermore, 36.6% were (53/145) White Brazilians, 45.5% (66/145) mixed-race Afro-Brazilians, 15.9% (23/145) Black Afro-Brazilians and 2.1% (3/145) East Asian Brazilians. Over half of the participants were in Social Class E, receiving <2× minimum wage (50.8%; 67/132). In total, 19.6% (27/138) of the study population were recipients of Bolsa Família (Table 1).

**Figure 3:**
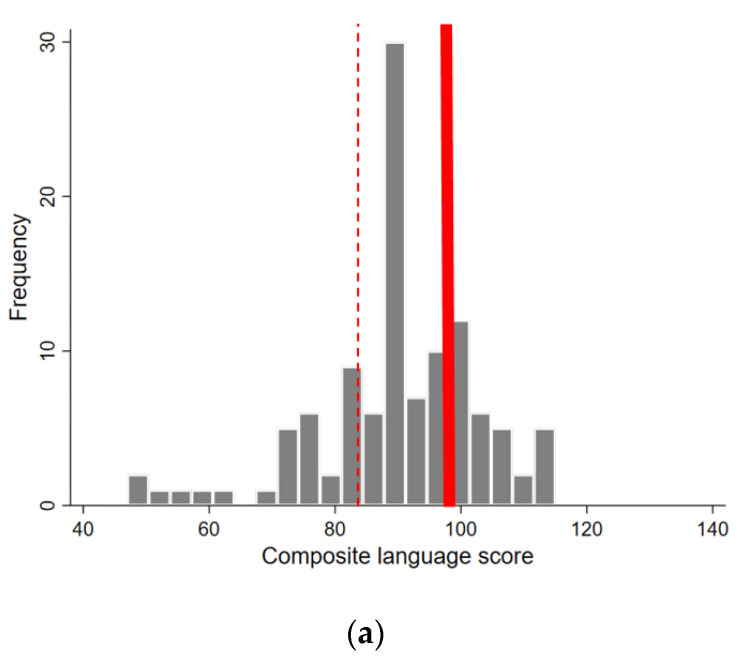

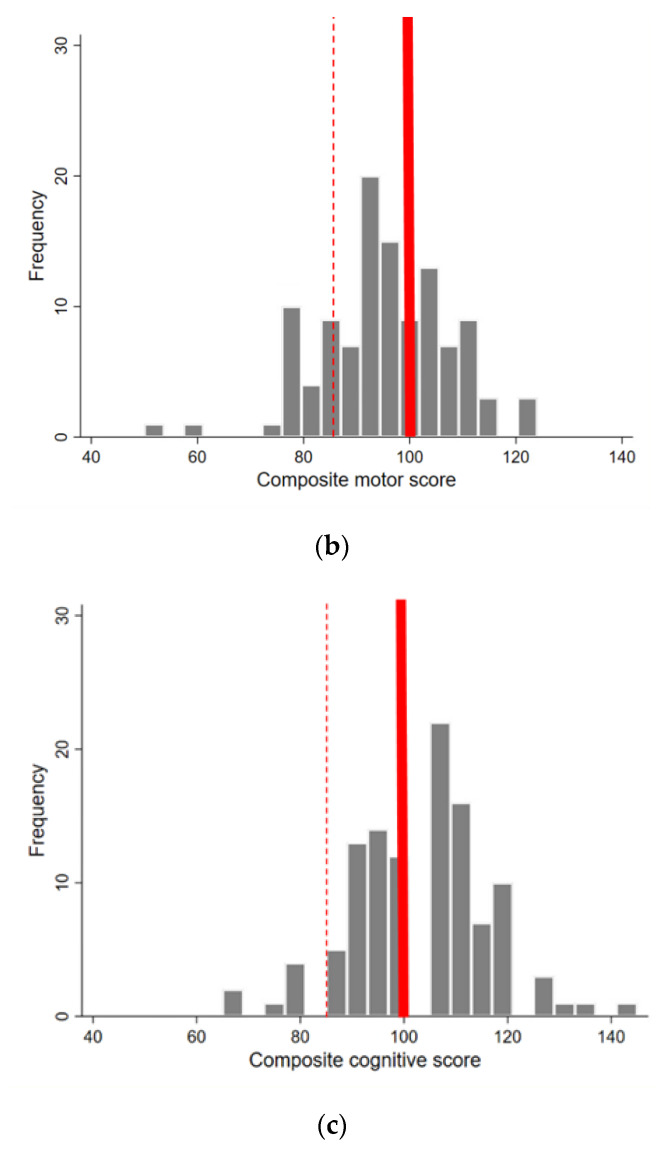
Histograms of composite language (**a**), motor (**b**) and cognitive (**c**) scores from Bayley-III assessments (thick red lines indicate the mean in a normative population (100) and dotted red lines indicate the threshold for developmental delay (at risk or severely delayed) at 1 or more SD below the mean (i.e., a score ≤85)).

There was a mean composite language score of 90.3 (SD ± 13.1), minimum and maximum of 47 and 115, a mean composite motor score 95.3 (SD ± 12.4), minimum and maximum of 50 and 124 and a mean composite cognitive score of 102.8 (SD ± 13.5), minimum and maximum of 65 and 145 (Figure 3). Among the 112 children with Bayley-III results, 25.9% (29/112) were at risk or severely delayed (i.e., 1 or more SD below the mean) for the composite language domain, 19.6% (22/112) were at risk or severely delayed for the composite motor domain and 10.7% (12/112) were at risk or severely delayed for the composite cognitive domain.

Crude analyses indicated strong evidence that children born into households with an income up to 2× minimum wage have 5.69 times (95% CI 2.43 to 13.33) the odds of having microcephaly compared to those born into a household with income over 2× minimum wage (Table 2). There was also a positive association between household participation in BF and the odds of microcephaly (OR, 95% CI: 2.55, 1.08 to 6.00). In addition, an increase in the level of maternal education, from primary to secondary school and to higher education, was strongly associated with a decrease in microcephaly odds (*p* < 0.001). The crude odds ratios for children with a mother with secondary school education and higher education compared with primary education were 0.37 (0.14, 0.99) and 0.12 (0.04, 0.38), respectively. Mothers who identified as Black Afro-Brazilian had the highest odds of having a child with microcephaly (OR, 95% CI: 3.55, 1.29 to 9.80), compared to the group with children born to mothers who identified as White Brazilian and East Asian Brazilian. In addition, there was evidence for a linear association between household crowding index (HCI) groups and the odds of microcephaly (OR, 95% CI: 1.79, 1.23 to 2.61) and no evidence for departures from linearity (*p* = 0.956).

Upon adjustment for child’s sex and household income, there was no statistical evidence of an association between race/ethnicity and microcephaly. The multivariate analysis indicated that having a household income of up to 2× minimum wage showed strong statistical evidence of an association with microcephaly (OR, 95% CI: 3.85, 1.43 to 10.37). Accounting for child’s sex and birthweight, lower maternal education was associated with an increase in microcephaly (*p* < 0.001). The adjusted odds for children with a mother with secondary school education and higher education compared with primary education were 0.33 (95% CI: 0.11 to 0.98) and 0.10 (95% CI: 0.01 to 0.36), respectively. After adjusting for child’s sex, maternal education and maternal parity, a linear trend was observed across the four household crowding index groups, such that each increase in household crowding index group (i.e., from least to most crowded) was associated with an 83% increase in the odds of microcephaly (OR, 95% CI: 1.83, 1.16 to 2.91) (Table 3). Consistent patterns of association were observed in sensitivity analyses including children without lab confirmation of prenatal ZIKV exposure (Table A2).

After adjusting for child sex, gestational age, maternal education, maternal race/ethnicity, household crowding index, maternal parity, previous miscarriage or abortion and birthweight, there was evidence of an association between household participation in Bolsa Família and a lower composite language score of −10.78 (95% CI: −19.87 to −1.69), a lower composite motor score of −10.45 (95% CI: −19.22 to −1.69) and a lower composite cognitive score of −17.20 (95% CI: −26.13 to −8.28) (Table 4). Bayley-III assessment scores did not appear to vary by other socioeconomic indicators in this study sample. Unadjusted estimates are presented in the appendices (Table A3).

## 4. Discussion

In a cohort of 163 infants with prenatal ZIKV exposure in Rio de Janeiro, Brazil, a consistent relationship between adverse neurodevelopmental outcomes and unfavourable socioeconomic indicators was observed. Specifically, these findings provide evidence of an association of microcephaly with lower household income, higher household crowding and lower maternal education. In line with these results, economically deprived children with prenatal ZIKV exposure also appeared to be at greater risk of delayed neurodevelopment during the early life course. Adjusted models provided statistical evidence of lower composite language, motor and cognitive scores in children whose families participated in the in the Bolsa Família social protection programme. Taken together, these findings reinforce the idea that early disadvantage can drive differential health and developmental outcomes [7].

Results from this study are consistent with previous research undertaken at the population level. An ecological analysis completed between 2015 and 2016 in Recife, Brazil, described a strong association between microcephaly from ZIKV infection and poor living conditions, such that only 2.0% of the microcephaly cases resided in the wealthiest districts [5]. Another ecological study conducted using socioeconomic and health status data from the five regions in Brazil reported a strong correlation between the distribution of ZIKV-related microcephaly cases and poverty as measured in an index (*p* < 0.0001) [20], suggesting the potential for co-acting socioeconomic factors in the microcephaly epidemic [21].

Adverse environmental conditions often cluster together in socially patterned ways [22]. People with low SEP are likely to live in adverse social circumstances, be of low birthweight and be exposed to poor diets [23]. A 1990–1991 cross-sectional study, investigating Aboriginal children under 2 years in Australia provided evidence that wasting was strongly associated with microcephaly on admission to a tertiary referral centre for diarrhoea, independent of intrauterine growth restriction and low birthweight. Low household income may drive food insecurity and thus malnutrition. Malnutrition, in important periods of intra- and extra-uterine development, could cause irreversible damage to intellectual potential and behaviour [24].

Often, where household crowding exists, neighbourhood overcrowding persists. The built environment in poor urban areas may also provide abundant habitats for mosquito proliferation through insufficient infrastructure [25]. In addition, housing can be seen as a key component of wealth as it often accounts for a large proportion of outgoings from income [26].

Education is a frequently used indicator of SEP with origins in the status domain of Weberian theory [26]. The variable of maternal education reflects mothers’ early life SEPs and captures their knowledge-related assets over the life course [26]. Inferences have been made in previous studies about how the underlying social environment, including low maternal education, may play a role in the development of neonatal microcephaly [27,28]. Two 2010 birth cohort studies conducted in Brazil concluded that low maternal schooling was consistently associated with microcephaly, suggesting that prior to the ZIKV epidemic, there may have been a silent endemic of microcephaly caused by other risk factors associated with poverty [28]. Crude analyses revealed a strong association between women who identified as Black Afro-Brazilian and having a child with microcephaly. After controlling for confounders, including household income, there was no statistical evidence of this association. This points to structural racism as a potential driver of neurodevelopmental disparities. Structural racism is defined by social epidemiologist, Nancy Krieger (2014) as “…ways in which societies foster [racial] discrimination… that in turn reinforce discriminatory beliefs, values, and distribution of resources” [29]. Many residents in Rio de Janeiro live in racialised and economically segregated areas of the city [30], which could be associated with health outcomes, including birth outcomes, as previously observed in the US context [31].

Furthermore, the findings from this study may be related to a lack of access to abortion services. Since abortion in Brazil is considered a crime against human life, except under exceptional circumstances, quantifying self-induced or unregulated abortion is extremely challenging [32]. Illegal options are available at a cost. Thus, one potential pathway for the outcomes observed is that those with lower household income may not have the means to pay for an abortion.

This investigation revealed lower composite language, motor and cognitive scores in children whose families were recipients of Bolsa Família. Whilst participation in Bolsa Família can be viewed as a proxy indicator for poverty as it is dependent on having a per capita monthly income ≤BRL 140 (US $35.00), it also indicates receipt of financial and social support. Those eligible for the programme must ensure compliance with selected activities, including schooling and vaccination for children and pre- and post-natal care for women [33]. This poverty-alleviating programme has the potential to improve poor health and development opportunities, as has been shown for diseases like leprosy [33,34]. An important concern in the current investigation may therefore be residual confounding. Thus, this warrants further investigation. Furthermore, eligibility assessments for this programme are made every two years; however, social circumstances may change over time. This highlights the challenges inherent in investigating social determinants of health without utilising a life course approach [22].

This investigation is a unique and important analysis. Whilst social determinants of ZIKV and CZS have been investigated primarily through ecological studies, this is the first study to describe the association of SEP at the individual level with microcephaly and delayed neurodevelopment following in utero exposure to ZIKV. Nevertheless, this study had important limitations. First, Rio de Janeiro presents a unique context of inequality, poverty, urban segregation and deficient infrastructure [35]. The results obtained from this study are therefore specific to this urban setting and thus may not be generalisable to rural communities in Brazil or indeed other urban environments outside of Brazil. Second, although a strength of this study is that it used stringent inclusion criteria and eligible infants were enrolled only if they had nucleic acid and/or serologic evidence of prenatal ZIKV exposure, it was not possible to confirm congenital ZIKV infection in all of the participating children. Third, the enrolment procedure may have introduced systematic error through selection bias. This dataset was biased towards children born with CZS, as women who were asymptomatic or who did not appear to have foetal abnormalities during pregnancy were not enrolled in the cohort. Whilst frequencies of outcomes are likely to be higher than the general population, the same selective forces within the study population that resulted in the outcomes of interest are expected to be similar across exposure groups. This is therefore unlikely to have distorted effect estimates. In addition, whilst the Unified Health System (Sistema Único de Saúde) has helped Brazil to progress towards universal health coverage, structural weaknesses as well as economic and political crises have resulted in disparities in access to effective care [36]. The poorest are less likely to frequent healthcare facilities and the wealthiest often utilise high-cost private clinics. Those of lower SEP not only experience access inequity but poorer knowledge of the full implications of ZIKV and reduced health-seeking behaviour [37]. Under-representation of the lowest and highest SEP categories may have resulted in different measurements of outcomes within these groups, though comparisons between them are still accurate. Fourth, if children did not appear to have microcephaly at birth, parents may have been reluctant to attend the hospital for further evaluations, as CZS is a highly stigmatising diagnosis [38]. This suggests likely attrition bias within the normocephalic group. If the participants with lower SEP who were lost to follow-up are at greater risk of neurodevelopmental delays, then the study will have underestimated the effect of low SEP. Furthermore, hospital visits are time-consuming and economic losses may occur following time off work. Fifth, self-reporting of the exposure variables may have resulted in non-differential social desirability bias, particularly with respect to reporting income, drug-taking and smoking. Since this would increase the similarity between the exposed and non-exposed, any true association between low SEP and the outcome measures would be attenuated. This is not likely to have been exacerbated by requirements in place to be a beneficiary of Bolsa Família, since decisions are based on data captured within the national administrative database, Cadastro Único para Programas Sociais [33]. Finally, sudden and unexpected disease outbreaks, such as the recent ZIKV epidemic, have erupted in settings with notable resource constraints [39,40]. Strategic decisions are thus required to optimise available resources but may lead to missing data, limited sample sizes and losses to follow-up [39]. Conducting analyses on clinical studies in these climates, as this study does, whilst challenging, provides important insight into novel and unknown disease patterns and global health problems.

## 5. Conclusions

This report provides new evidence of the link between social determinants and the risk of microcephaly and delayed childhood neurodevelopment following in utero exposure to ZIKV. These findings suggest that targeting interventions, such as culturally appropriate and economically viable vector control measures, to socioeconomically marginalised groups may aid in reducing the disease burden of ZIKV in the case of a future epidemic. More broadly, this research also reflects the need in ZIKV research to expand the focus from a strictly biomedical paradigm of health and developmental outcomes, in which diagnosis and treatment focus on an individual’s biology, to an integrated approach that addresses social factors [41]. Further research will be valuable for delineating the mechanisms by which low SEP may exert corrosive effects following prenatal exposure to ZIKV.

## Figures and Tables

**Table 1:** Baseline distribution of selected cohort characteristics (*n* = 163).

Variable	Category	Total, No.	No. (col%)/ Median (IQR)
Characteristics of children			
Sex	Female	163	84 (51.5%)
	Male		79 (48.5%)
Gestational age (weeks)		163	38 (38–40)
Birthweight (g)		163	3060 (2675–3420)
Age at last Bayley-III test (months)		112	19.6 (12.8–36.0)
Characteristics of mothers			
Age at enrolment (years)	Median (IQR)	126	30.8 (23.6–34.7)
Educational level	Primary school, including partial	147	22 (15.0%)
	Secondary school, including partial		77 (52.4%)
	Higher education, including partial		48 (32.7%)
Race/ethnicity	White Brazilians	145	53 (36.6%)
	Mixed-race Afro-Brazilians		66 (45.5%)
	Black Afro-Brazilians		23 (15.9%)
	East Asian Brazilians		3 (2.1%)
Parity	≤1	151	81 (53.6%)
	2		50 (33.1%)
	3+		20 (13.3%)
Previous miscarriage or abortion	No	143	117 (81.8%)
	Yes		26 (19.2%)
Trimester of pregnancy with rash	First	130	56 (43.1%)
	Second		49 (37.7%)
	Third		25 (19.2%)
Smoking during pregnancy	No	145	139 (95.9%)
	Yes		6 (4.1%)
Drug use during pregnancy	No	146	138 (94.5%)
	Yes		8 (5.5%)
Occupational exposure to toxic products during pregnancy	No	143	111 (77.6%)
	Yes		32 (22.4%)
Characteristics of household			
Monthly income (relative to 2019 minimum wage of BRL 998)	Class A: >20× minimum wage	132	2 (1.5%)
	Class B: 10–20× minimum wage		7 (5.3%)
	Class C: 4–10× minimum wage		18 (13.6%)
	Class D: 2–4× minimum wage		38 (28.8%)
	Class E: <2× minimum wage		67 (50.8%)
Participation in Bolsa Família	No	138	111 (80.4%)
	Yes		27 (19.6%)
Household crowding index	<0.50	136	38 (27.9%)
	0.50–0.75		46 (33.8%)
	0.75–1.00		33 (24.3%)
	1.00+		19 (14.0%)

**Table 2:** Baseline distribution of child, maternal and household characteristics with crude odds ratios of microcephaly in children exposed to ZIKV in utero (*n* = 163).

Variable	Category	Number of Children Exposed to ZIKV In Utero	No. (Row %) of Exposed Children with Microcephaly	Crude Odds Ratio (95% CI)	*p* Value */**
Characteristics of children					
Sex	Female	84	27 (32.1%)	1	0.808
	Male	79	24 (30.4%)	0.92 (0.47, 1.79)	
Characteristics of mothers					
Educational level	Primary school, including partial	22	13 (59.1%)	1	<0.001
	Secondary school, including partial	77	27 (35.1%)	0.37 (0.14, 0.99)	
	Higher education, including partial	48	7 (14.6%)	0.12 (0.04, 0.38)	
	Missing	16	4 (25.0%)		
Race/ethnicity	White Brazilian and East Asian Brazilian	56	15 (26.8%)	1	0.038
	Mixed-race Afro-Brazilian	66	19 (28.8%)	1.10 (0.50, 2.45)	
	Black Afro-Brazilian	23	13 (56.5%)	3.55 (1.29, 9.80)	
	Missing	18	4 (22.2%)		
Parity	≤1	81	24 (29.6%)	1	0.887
	2	50	16 (32.0%)	1.12 (0.52, 2.39)	
	3+	20	7 (35.0%)	1.28 (0.45, 3.60)	
	Missing	12	4 (33.3%)		
Previous miscarriage or abortion	No	117	37 (31.6%)	1	0.506
	Yes	26	10 (38.5%)	1.35 (0.56, 3.26)	
Trimester of pregnancy with rash	First	56	31 (55.4%)	1	<0.001
	Second	49	3 (6.1%)	0.05 (0.01, 0.19)	
	Third	25	2 (8.0%)	0.07 (0.02, 0.33)	
	Missing	33	15 (45.5%)		
Smoking during pregnancy	No	139	44 (31.7%)	1	0.363
	Yes	6	3 (50.0%)	2.16 (0.42, 11.13)	
	Missing	18	4 (22.2%)		
Drug use during pregnancy	No	138	44 (31.9%)	1	0.744
	Yes	8	3 (37.5%)	1.28 (0.29, 5.61)	
	Missing	17	4 (23.5%)		
Occupational exposure to toxic products during pregnancy	No	111	37 (33.3%)	1	0.824
	Yes	32	10 (31.3%)	0.90 (0.39, 2.12)	
	Missing	20	4 (20.0%)		
Characteristics of household					
Income per month (relative to minimum wage)	Classes A, B, C and D: >2× min wage	65	9 (13.8%)	1	<0.001
	Class E: <2× min wage	67	32 (47.8%)	5.69 (2.43, 13.33)	
	Missing	31	10 (32.3%)		
Participation in Bolsa Família	No	111	33 (29.7%)	1	0.033
	Yes	27	14 (51.9%)	2.55 (1.08, 6.00)	
	Missing	25	4 (16.0%)		
Household crowding index	<0.5	38	7 (18.4%)	1	0.081
	0.5–0.75	46	13 (28.3%)	1.74 (0.62, 4.94)	
	0.75–1.0	33	13 (39.4%)	2.88 (0.98, 8.45)	
	1.0+	19	11 (57.9%)	6.09 (1.79, 20.74)	
	Missing	27	7 (25.9%)		

* *p* values do not include missing data categories. ** The likelihood ratio test was used to assess the strength of the evidence of the association between exposure variables and outcomes.

**Table 3:** Multivariate associations of socioeconomic indicators with microcephaly cases.

Variable	Category	Adjusted Odds Ratio (95% CI)	*p* Value *
Maternal educational level ^a^ *n* = 147	Primary school, including partial	1	<0.001
	Secondary school, including partial	0.33 (0.11, 0.98)	
	Higher education, including partial	0.10 (0.03, 0.36)	
Maternal race/ethnicity ^b^ *n* = 129	White Brazilian and East Asian Brazilian	1	0.439
	Mixed-race Afro-Brazilian	0.89 (0.35, 2.27)	
	Black Afro-Brazilian	1.79 (0.55, 5.86)	
Household monthly income ^c^ *n* = 122	Classes A, B, C and D: > 2× min wage	1	0.006
	Class E: <2× min wage	3.85 (1.43, 10.37)	
Household participation in Bolsa Família ^d^ *n* = 135	No Yes	1 1.74 (0.69, 4.37)	0.239
Household crowding index ^e^ *n* = 129	Household crowding index groups (<0.5, 0.5–0.75, 0.75–1.0, 1.0+)	1.83 (1.16, 2.91)	0.008
	

^a^ adjusted for child’s sex, birthweight; ^b^ adjusted for child’s sex, household income; ^c^ adjusted for child’s sex, household crowding index, maternal education; ^d^ adjusted for child’s sex, maternal education, previous miscarriage or abortion; ^e^ adjusted for child’s sex, maternal education, maternal parity; * The likelihood ratio test was used to assess the strength of the evidence of the association of exposure variables and outcomes.

**Table 4:** Adjusted estimated differences in Bayley-III assessment scores according to risk factors, in the normocephalic study sample (n = 112).

		Composite Language		Composite Motor		Composite Cognitive	
Variables	Categories	Adjusted Estimated Difference in Composite Scores (95% CI)	*p* Value *	Adjusted Estimated Difference in Composite Scores (95% CI)	*p* Value *	Adjusted Estimated Difference in Composite Scores (95% CI)	*p* Value *
Maternal educational level ^a^ *n* = 83	Primary school, including partial	(Reference)	0.821	(Reference)	0.975	(Reference)	0.200
	Secondary school, including partial	3.31(−7.96, 14.57)		−0.21 (−10.69, 10.26)		−4.19 (−15.09, 6.71)	
	Higher education, including partial	3.00 (−8.61, 14.57)		0.51 (−10.27, 11.29)		1.80 (−9.41, 13.01)	
Maternal race/ethnicity ^b^ *n* = 76	White Brazilians and East Asian Brazilians	(Reference)	0.945	(Reference)	0.253	(Reference)	0.262
	Mixed-race Afro-Brazilians	0.88 (−6.29, 8.05)		2.86 (−3.97, 9.69)		4.56 (−2.33, 11.46)	
	Black Afro-Brazilians	1.53 (−10.67, 13.73)		8.51 (−3.11, 20.13)		6.38 (−5.35, 18.11)	
Household monthly income ^c^ *n* = 78	Classes A, B, C and D: >2× min wage	(Reference)	0.411	(Reference)	0.594	(Reference)	0.905
	Class E: <2× min wage	2.77 (−4.58, 10.13)		1.72 (−5.35, 8.80)		0.41 (−7.12, 7.95)	
Household participation in Bolsa Família ^d^ *n* = 80	No Yes	(Reference) −10.78 (−19.87, −1.69)	0.011	(Reference) −10.45 (−19.22, −1.69)	0.011	(Reference) −17.20 (−26.13, −8.28)	<0.001
Household crowding index ^e^ *n* = 76	Linear trend across four household crowding index groups (<0.5, 0.5–0.75, 0.75–1.0, 1.0+)	−1.45 (−5.11, 2.20)	0.380	1.44 (−2.05, 4.92)	0.363	2.79 (−0.72, 6.30)	0.082

^a^ adjusted for child sex, gestational age, maternal race/ethnicity, household income, household participation in Bolsa Família, household crowding index. ^b^ adjusted for child sex, gestational age, maternal education, household income, household participation in Bolsa Família, household crowding index, maternal parity, previous miscarriage or abortion, birthweight. ^c^ adjusted for child sex, gestational age, maternal education, maternal race/ethnicity, household crowding index, maternal parity, previous miscarriage or abortion, birthweight. ^d^ adjusted for child sex, gestational age, maternal education, maternal race/ethnicity, household crowding index, maternal parity, previous miscarriage or abortion, birthweight. ^e^ adjusted for child sex, gestational age, maternal education, maternal race/ethnicity, household income, household participation in Bolsa Família, maternal parity, previous miscarriage or abortion, birthweight. * The likelihood ratio test was used to assess the strength of the evidence of the association between exposure variables and outcomes.

## Appendix A

**Table A1:** Missingness patterns with percentages of missing data for each variable and baseline information stratified into two groups: cases with complete data and cases with missing data (*n* = 163).

Variable	Category	Total, No.	% Non-Missing Data	Cases with Complete Data No. (%) *n* = 83	Cases with Missing Data No. (%)	*p* Value
Characteristics of children						
Child sex	Female	163	100%	41	43	0.578
	Male			42	37	
Characteristics of mothers						
Maternal educational level	Primary school, including partial	147	90%	16	6	0.119
	Secondary school, including partial			38	39	
	Higher education, including partial			29	10	
Maternal race/ethnicity	White Brazilian and East Asian Brazilian	145	89%	34	22	0.645
	Mixed-race Afro-Brazilian			35	31	
	Black Afro-Brazilian			14	9	
Maternal parity	≤1	151	93%	41	40	0.291
	2			32	18	
	3+			10	10	
Previous miscarriage or abortion	No	143	88%	69	48	0.632
	Yes			14	12	
Trimester of pregnancy with rash	First	130	80%	39	17	0.443
	Second			30	19	
	Third			14	11	
Did the mother smoke during pregnancy	No	145	89%	79	60	0.634
	Yes			4	2	
Did the mother use drugs during pregnancy	No	146	90%	80	58	0.256
	Yes			3	5	
Occupational exposure to toxic products during pregnancy	No	143	88%	65	46	0.816
	Yes			18	14	
Characteristics of household						
Household monthly income (relative to minimum wage of BRL998)	Class A: >20× minimum wage	132	81%	38	29	0.684
	Class B: 10–20× minimum wage			5	2	
	Class C: 4–10× minimum wage			12	6	
	Class D: 2–4× minimum wage			26	12	
	Class E: <2× minimum wage			38	29	
Household participation in Bolsa Família (government cash transfer scheme)	No	138	85%	67	61	0.513
	Yes			16	11	
Household crowding index (individuals in the house / rooms in the house)	<0.5	136	83%	23	15	0.584
	0.5–0.75			25	21	
	0.75–1.0			23	10	
	1.0+			12	7	

## Appendix B

**Table A2:** Multivariate associations of socioeconomic indicators with microcephaly cases in children with and without qRT-PCR or IgM confirmation with suspected prenatal ZIKV exposure (*n* = 286).

Variable	Category	Adjusted Odds Ratio (95% CI)	*p* Value *
Maternal educational level ^a^ *n* = 240	Primary school, including partial	1	<0.001
	Secondary school, including partial	0.71 (0.33, 1.54)	
	Higher education, including partial	0.22 (0.09, 0.55)	
Maternal race/ethnicity ^b^ *n* = 212	White Brazilians and East Asian Brazilians	1	0.526
	Mixed-race Afro-Brazilians	0.83 (0.34, 1.99)	
	Black Afro-Brazilians	0.67 (0.34, 1.35)	
Household monthly income (relative to minimum wage) ^c^ *n* = 204	Classes A, B, C and D: >2× min wage	1	0.004
	Class E: <2× min wage	2.71 (1.36, 5.43)	
Household participation in Bolsa Família (government cash transfer) scheme ^d^ *n* = 224	No	1	0.371
	Yes	1.35 (0.70, 2.62)	
Household crowding index (individuals in the house/rooms in the house) ^e^ *n* = 207	Linear trend across four household crowding index groups (<0.5, 0.5–0.75, 0.75–1.0, 1.0+)	1.48 (1.06, 2.09)	0.021

^a^ adjusted for child’s sex and birthweight. ^b^ adjusted for child’s sex and household income. ^c^ adjusted for child’s sex, household crowding index and maternal education. ^d^ adjusted for child’s sex, maternal education and previous miscarriage or abortion. ^e^ adjusted for child’s sex, maternal education and maternal parity * The likelihood ratio test was used to assess the strength of the evidence of an association between exposure variables and outcomes.

## Appendix C

**Table A3:** Unadjusted estimated differences in Bayley-III assessment scores according to risk factors, in the normocephalic study sample (*n* = 112).

Variable	Category	No. (Col %) of Children Taking Bayley-III Tests	Unadjusted Estimated Difference in Language Scores (95% CI)	*p* Value */**	Unadjusted Estimated Difference in Motor Scores (95% CI)	*p* Value */**	Unadjusted Estimated Difference in Cognitive Scores (95% CI)	*p* Value */**
Characteristics of children								
Sex	Female	57 (50.9%)	(Reference)	0.429	(Reference)	0.937	(Reference)	0.752
	Male	55 (49.1%)	−1.97 (−6.90, 2.96)		−0.19 (−4.84, 4.47)		0.81 (−4.28, 5.90)	
Characteristics of mother								
Educational level	Primary school, including partial	9 (8.0)	(Reference)	0.459	(Reference)	0.94	(Reference)	0.139
	Secondary school, including partial	50 (44.6%)	2.34 (−7.00, 11.69)		−0.87 (−9.84, 8.10)		−3.63 (−12.92, 5.65)	
	Higher education, including partial	41 (36.6%)	5.03 (−4.47, 14.53)		0.01 (−9.11, 9.13)		1.79 (−7.65, 11.23)	
	Missing	12 (10.7%)						
Race/ethnicity	White Brazilians and East Asian Brazilians	41 (36.6%)	(Reference)	0.55	(Reference)	0.369	(Reference)	0.947
	Mixed-race Afro-Brazilians	47 (42.0%)	−2.78 (−8.32, 2.77)		3.67 (−1.62, 8.96)		0.74 (−4.90, 6.38)	
	Black Afro-Brazilians	10 (8.9%)	−3.51 (−12.67, 5.64)		3.42 (−5.31, 12.15)		1.29 (−8.−1, 10.60)	
	Missing	14 (12.5%)						
Parity	≤1	57 (50.9%)	(Reference)	0.211	(Reference)	0.007	(Reference)	0.566
	2	34 (30.4%)	0.66 (−4.97, 6.30)		−7.32 (−12.49, −2.16)		0.93 (−4.92, 6.77)	
	3+	13 (11.6%)	7.10 (−0.89, 15.10)		3.04 (−4.28, 10.37)		4.47 (−3.82, 12.76)	
	Missing	8 (7.1%)						
Previous miscarriage or abortion	No	80 (71.4%)	(Reference)	0.62	(Reference)	0.552	[Reference)	0.932
	Yes	16 (14.3%)	1.76 (−5.27, 8.79)		−2.04 (−8.81, 4.74)		−0.31 (−7.56, 6.93)	
	Missing	16 (14.3%)						
Trimester of pregnancy with rash	First	25 (22.3%)	(Reference)	0.138	(Reference)	0.037	(Reference)	0.523
	Second	46 (41.1%)	−0.30 (−6.81, 6.21)		−6.25 (−12.26, −0.23)		−0.39 (−6.94, 6.16)	
	Third	23 (20.5%)	−6.56 (−14.13, 1.01)		−8.72 (−15.71, −1.73)		−3.87 (−11.48, 3.75)	
	Missing	18 (16.1%)						
Smoke during pregnancy	No	96 (85.0%)	(Reference)	1	(Reference)	−0.409	(Reference)	0.944
	Yes	3 (2.7%)	0.00 (−15.21, 15.22)		−5.63 (−19.12, 7.86)		−0.54 (−15.87, 14.79)	
	Missing	14 (12.4%)						
Drug use during pregnancy	No	95 (84.8%)	(Reference)	0.121	(Reference)	0.797	(Reference)	0.818
	Yes	3 (2.7%)	−9.30 (−21.09, 2.49)		1.47 (−9.86, 12.81)		−1.39 (−13.38, 10.60)	
	Missing	14 (12.5%)						
Occupational exposure to toxic products during pregnancy	No	74 (66.1%)	(Reference)	0.735	(Reference)	0.404	(Reference)	0.611
	Yes	22 (19.6%)	1.09 (−5.26, 7.44)		2.54 (−3.49, 8.57)		1.65 (−4.78, 8.09)	
	Missing	16 (14.3%)						
Characteristics of household								
Monthly income (relative to minimum wage)	Classes A, B, C and D: > 2× min wage	56 (50.0%)	(Reference)	0.169	(Reference)	0.895	(Reference)	0.460
	Class E: < 2× Min wage	35 (31.3%)	−4.03 (−9.79, 1.74)		−0.36 (−5.84, 5.12)		−2.16 (−7.95, 3.63)	
	Missing	21 (18.8%)						
Participation in Bolsa Família	No	78 (69.6%)	(Reference)	0.078	(Reference)	0.039	(Reference)	0.019
	Yes	13 (11.6%)	−7.05 (−14.92, 0.81)		−7.79 (−15.20, −0.39)		−9.42 (−17.23, −1.61)	
	Missing	21 (18.8%)						
Household crowding index	<0.5	31 (27.7%)	(Reference)	0.76	(Reference)	0.212	(Reference)	0.280
	0.5–0.75	33 (29.5%)	−0.74 (−7.33, 5.85)		5.17 (−0.99, 11.34)		5.97 (−0.66, 12.59)	
	0.75–1.0	20 (17.9%)	−0.34 (−7.90, 7.22)		0.94 (−6.13, 8.00)		3.72 (−3.88, 11.31)	
	1.0+	8 (7.1%)	−5.57 (−16.02, 4.89)		7.89 (−1.88, 17.65)		7.22 (−3.29, 17.72)	
	Missing	20 (17.8%)						

* *p* values do not include missing data categories ** The likelihood ratio test was used to assess the strength of the evidence of an association between exposure variables and outcomes.
